# Utilization of Training for Nurses in the Prevention of Adolescent Violence Risk Behaviors: Protocol for a Scoping Review

**DOI:** 10.2196/89576

**Published:** 2026-08-10

**Authors:** Sri Maryatun, Intansari Nurjannah, Ariani Arista Putri Pertiwi

**Affiliations:** 1 Doctoral Program, Faculty of Medicine, Public Health and Nursing, Universitas Gadjah Mada, Palembang City, South of Sumatera, Indonesia; 2 Nursing Study Program, Faculty of Medicine, Universitas Sriwijaya, South of Sumatera, Indonesia; 3 Departement of Mental Health and Community Nursing, Faculty of Medicine, Public Health and Nursing, Universitas Gadjah Mada, Yogyakarta, Indonesia; 4 Department of Basic and Emergency Nursing, Faculty of Medicine, Public Health and Nursing, Universitas Gadjah Mada, Jl. Farmako, Sekip UtaraYogyakarta, 55281, Indonesia, +6287878208878

**Keywords:** adolescent violence, aggressive behaviour, mental health, nurse education, prevention, scoping review

## Abstract

**Background:**

Adolescent violence is a significant public health issue that negatively affects the psychological well-being and social functioning of adolescents. These tendencies generally evolve from problems in anger management and stress tolerance related to inherent or external contributors including familial background, interactions with friends, and school atmosphere. Nurses, particularly in primary health care settings, are strategically positioned to contribute to violence prevention through early identification, intervention, and capacity-building programs for adolescents. However, evidence regarding the implementation of nurse-led violence prevention training, including its effectiveness, training approaches, and contextual influencing factors, remains limited.

**Objective:**

This scoping review will map the nature, content, approach, and effectiveness of educational interventions developed for nurses to prevent violent behavior among adolescent learners in health services.

**Methods:**

The protocol adheres to the Joanna Briggs Institute guidelines and the Arksey and O’Malley framework, including identifying the research question, searching for relevant studies, selecting eligible studies, charting the data, collating and summarizing the findings, and consulting experts. This review will be reported in accordance with the PRISMA extension for Scoping Reviews (PRISMA-ScR) guidelines. Literature searches will be conducted in PubMed, Scopus, ProQuest, and the Cochrane Library, along with gray literature sources including Google Scholar and GreyNet. Studies published in English that focus on educational or training interventions aimed at improving nurses’ capacity to prevent adolescent violence will be included, whereas studies unrelated to nurse-focused interventions or adolescent violence prevention will be excluded. Reviewers will independently conduct study selection and data extraction using a standardized extraction form. Extracted data will include study characteristics, intervention types, implementation strategies, outcomes, and contextual factors. The findings will be synthesized descriptively through narrative summaries and tabulated evidence mapping.

**Results:**

Literature searches across the selected electronic databases were initiated in March 2025 using predefined search terms. Title and abstract screening, as well as preliminary appraisal of eligible studies, were conducted in May 2026 following the Joanna Briggs Institute methodology and PRISMA-ScR guidelines. Full-text screening, data extraction, and thematic analysis are currently ongoing and are expected to be completed by July 2026. The findings of this scoping review are expected to be submitted to a journal by September 2026.

**Conclusions:**

This scoping review will provide a comprehensive evidence map of available training programs, implementation strategies, barriers to utilization, and their impact on nurses’ knowledge, attitudes, and skills related to adolescent violence risk behaviors. The findings are expected to support the scientific foundation for the development of the training model of the Prevention of Adolescent Violence-Risk Behavior (*Keterampilan Pencegahan Risiko Perilaku Kekerasan Remaja* [Kepolar]) in primary health care and inform policymaking to strengthen health professionals’ capacity in preventing adolescent violent behavior.

## Introduction

### Background

Adolescence is a unique life stage, associated with extensive biological, cognitive, and social-emotional transitions [1]. Adolescents also begin to move from an egocentric mode of thought to a more abstract and introspective kind [2]. Unfortunately, immature cognitive development tends to make adolescents “thoughtless,“ “irritable,” and “reckless,” accompanied by increases in the risk of violent behaviors as well [1]. Emotional dysregulation and the obsessive quest for autonomy in a context of insufficient cognitive maturity render adolescents susceptible to inappropriate outbursts of anger [3,4].

The World Health Organization defines violence as the intentional use of physical force or power that may result in injury, psychological harm, developmental disruption, or death [5]. Adolescent violent behavior may manifest in various forms, including physical aggression, verbal aggression, threatening behavior, bullying perpetration, fighting, emotional violence, and other externalizing behaviors [6,7]. These behaviors are associated with serious short- and long-term consequences, including physical injury, substance misuse, academic problems, criminal involvement, impaired social functioning, and mental health disorders [8,9]. Violence among adolescents also contributes substantially to the burden experienced by families, schools, health care systems, and communities [10].

Recent evidence indicates increasing trends in peer aggression, physical violence, emotional violence, and other forms of externalizing behavior among adolescents worldwide [11]. A UNESCO (United Nations Educational, Scientific and Cultural Organization) report estimated that approximately one-third of adolescents aged 11‐15 years have experienced involvement in bullying or peer violence within school environments [12]. Over the past year, one-third of students (36%) aged 13‐15 years were involved in a physical fight with another student, and almost one in three (32%) had been physically attacked at least once [13]. A study in Indonesia, the National Survey on the Life Experiences of Children and Adolescents [14], found two in three children and adolescents have experienced violence at least once in their lives, with this figure increasing [15]. These findings highlight the urgent need for early identification, prevention, and intervention strategies targeting adolescents at risk of violent behavior, particularly within community and primary health care settings [16].

Adolescent violent behavior is influenced by multiple interacting internal and external factors. Internal factors include age, sex, impulsivity, emotional regulation, psychological conditions, coping abilities, and spirituality [17,18]. External factors include parenting style, family conflict, peer influence, school climate, exposure to community violence, and broader social environments [19,20]. Conversely, protective factors such as family connectedness, empathy, religiosity, social support, and positive school engagement may reduce the likelihood of violent behavior [21,22]. Understanding these multidimensional influences is important for health care professionals, particularly nurses, in supporting early risk identification, prevention planning, and violence management among adolescents [23].

Health care professionals, especially nurses working in primary health care and community settings, play an important role in preventing and managing adolescent violent behavior [24]. Nurses are often among the first health care providers to identify behavioral problems, aggression, emotional dysregulation, and psychosocial risks in adolescents [25]. Their roles may include early screening and risk assessment, psychoeducation, counseling, emotional regulation support, family involvement, crisis management, and referral coordination. Effective nursing interventions may contribute to reducing aggressive behavior and improving psychosocial and behavioral outcomes among adolescents [26].

Despite the important role of nurses, previous studies indicate that many nurses report insufficient preparedness, limited confidence, and inadequate competencies in managing adolescents with violent or aggressive behavior [27-29]. Limited training in adolescent mental health, violence prevention, behavioral management, and psychosocial intervention may reduce nurses’ ability to provide early detection and appropriate interventions in health care settings [30]. In Indonesia, these challenges may be more pronounced because mental health services in primary health care settings (*puskesmas*) still predominantly focus on severe mental disorders rather than preventive interventions targeting adolescent behavioral risks [31].

Recent studies suggest that education and training programs for health care professionals, including nurses, may improve confidence, preparedness, communication skills, risk assessment abilities, and management of violent or aggressive behaviors among adolescents [26]. Interactive approaches such as role-playing, simulation, case-based learning, and de-escalation training have been associated with improved competency development and more sustainable behavioral outcomes [32]. However, most violence prevention interventions have primarily focused on school-based or community-based youth programs, whereas evidence specifically examining nurse-targeted training for adolescent violence prevention and management remains limited [33]. Existing studies also commonly position nurses as facilitators or referral agents within broader community or school health initiatives rather than as primary targets of structured competency-based training programs [34].

Implementation of violence prevention training programs is influenced not only by individual competencies but also by institutional and systemic support, including operational guidelines, referral pathways, leadership support, protected intervention time, and intersectoral collaboration [35]. Although adolescent violence may occur across school and community environments, this scoping review specifically focuses on nurse training interventions in the Prevention of Adolescent Violence Risk-Behavior (*Keterampilan Pencegahan Risiko Perilaku Kekerasan Remaja* [Kepolar]) program implemented within health care and primary health care contexts.

Effective prevention and management of adolescent violent behavior require multidimensional approaches addressing individual, interpersonal, family, community, and societal factors, as described in the social ecological model [36,37]. Nursing interventions and training programs may also be informed by frameworks such as King’s Goal Attainment Theory [38] and Bronfenbrenner’s Ecological Systems Theory [39], which emphasize interactions among adolescents, health care providers, families, and surrounding environments. These theoretical perspectives may support the development of structured nurse training models for adolescent violence prevention within primary health care settings.

Current evidence related to violence prevention interventions remains methodologically heterogeneous, with many studies characterized by small sample sizes, inconsistent outcome measures, and limited reporting transparency. Existing evidence on nurse training interventions for adolescent violence prevention has also not been comprehensively mapped regarding theoretical frameworks, intervention components, implementation strategies, and reported outcomes. Therefore, a scoping review is needed to systematically identify, map, and synthesize available evidence regarding nurse training interventions for adolescent violence prevention and management within primary health care settings.

### Objective

This scoping review protocol seeks to offer a more structured and methodical approach for identifying young people who behave violently and ensuring they are referred to nurse education programs so that they may benefit from interventions provided therein. Our protocol is designed to make sure that the review procedure is carried out systematically and reproducibly, thereby reducing any possible bias and increasing methodological transparency as well as scientific trustworthiness.

The purpose of this review is to create an evidence-based training model that can be used by nurses in primary health care centers (*puskesmas*) to prevent adolescent violent behavior. In particular, this scoping review aims to discern and map the current scientific evidence on training for nurses in adolescent violence prevention regarding theoretic frameworks, the main elements of interventions, and intervention delivery strategies and effects. Finally, risk and protective factors related to adolescent violent behavior will be discussed in this review, as well as the instruments adopted for measuring nurses’ knowledge, attitudes, skills, and self-efficacy. It will offer further evidence of training interventions’ impact on these competences.

## Methods

This scoping review protocol follows the methodological guidance developed by Arksey and O’Malley [40], further refined by Levac et al [41], and aligns with the Joanna Briggs Institute methodology for scoping reviews. The review will also be reported in accordance with the PRISMA-ScR (Preferred Reporting Items for Systematic Reviews and Meta-Analyses extension for Scoping Reviews) guidelines. A systematic and iterative review process will be undertaken to comprehensively map existing evidence regarding training and educational interventions for nurses in the prevention of adolescent violence risk behaviors.

The review process will consist of six stages: (1) identifying the research questions; (2) developing eligibility criteria; (3) systematically identifying and selecting relevant studies; (4) charting and extracting data using a standardized extraction form; (5) collating, summarizing, and reporting the findings through descriptive narrative synthesis and evidence mapping; and (6) conducting expert consultation to strengthen the interpretation and applicability of the findings. We will conduct literature searches in PubMed, Scopus, ProQuest, and the Cochrane Library, as well as gray literature sources including Google Scholar and GreyNet. Two independent reviewers will perform study screening and data extraction, with disagreements resolved through discussion or consultation with a third reviewer when necessary.

### Protocol Registration and Reporting Information

This protocol has been registered in the Open Science Framework. The final report will be prepared in accordance with the PRISMA-ScR checklist to ensure transparency and completeness throughout all stages of the review.

### Research Questions

This scoping review seeks to map and synthesize the existing evidence regarding nurse training programs for the prevention of adolescent violent behavior in primary health care settings.

The main research question guiding this review is “What evidence exists regarding training programs for nurses in the prevention of adolescent violent behavior in primary health care settings?”

The review will further address the following subquestions:

1. What risk and protective factors associated with adolescent violent behavior are reported in the literature?
2. What types of training or educational programs for nurses related to adolescent violence prevention have been described?
3. What theoretical frameworks, intervention components, and delivery strategies are used in these training programs?
4. What instruments or assessment tools have been used to evaluate nurses’ knowledge, attitudes, skills, self-efficacy, or competencies related to adolescent violence prevention?
5. What outcomes or reported impacts of nurse training programs related to adolescent violence prevention have been described in the literature?

### Eligibility Criteria

The eligibility criteria for this scoping review were developed using the Population-Concept-Context (PCC) framework recommended by the Joanna Briggs Institute for scoping reviews (Table 1). The population of interest includes nurses and nursing personnel involved in providing care for adolescents who exhibit violent behavior. The concept focuses on violence prevention training interventions designed to enhance nurses’ capacity in managing adolescent violent behavior. These interventions may include educational programs, de-escalation training, communication skills training, simulation-based learning, and other strategies aimed at improving nurses’ knowledge, attitudes, competencies, confidence, preparedness, self-efficacy, and experiences in managing violent behavior among adolescents (Textbox 1). Comparators, where applicable, may include usual care, routine nursing practice without specific violence prevention training, health care services without active nurse involvement in violence prevention, or pre–post comparisons evaluating the effects of training interventions. The context of this review encompasses health care settings, community services, school-based health services, and primary health care facilities, including public health centers (*puskesmas*).

**Table 1.** Population-Concept-Context framework.

PCC	Description
Population	Nurses or nursing personnel involved in adolescent violence prevention and management
Concept	Training programs, educational interventions, simulation-based learning, de-escalation training, communication training, and competency development related to adolescent violence prevention
Context	Primary health care settings, community health care services, school health services, and other health care settings where nurses provide adolescent health services

Textbox 1. **Inclusion and exclusion criteria.**

**Inclusion criteria**

- Studies involving nurses or nursing personnel in training or educational interventions related to adolescent violence prevention or management
- Studies conducted in health care, community, or school health settings
- Studies describing theoretical frameworks (eg, social ecological model, goal attainment theory, or other relevant models)
- Qualitative, quantitative, mixed methods studies, reviews, and relevant gray literature
- Publications in English or Indonesian
- Studies published between 2000 and 2024

**Exclusion criteria**

- Studies focused exclusively on psychiatric disorder treatment without relevance to violence prevention or risk behavior management
- Studies without involvement of nurses or nursing personnel
- Editorials, opinion pieces, commentaries, and conference abstracts without full text
- Studies without accessible full text

The review will consider qualitative, quantitative, and mixed methods studies, as well as review articles and relevant gray literature published in English or Indonesian between 2000 and 2024. Editorials, commentaries, opinion papers, conference abstracts without full text, and studies not related to violence prevention training for nurses in the context of adolescent violent behavior will be excluded. Studies eligible for inclusion are those that discuss violence prevention training, intervention programs, or related educational strategies for nurses in the context of adolescent violent behavior management. Editorials, commentaries, opinion papers, conference abstracts without full text, and studies not relevant to violence prevention training for nurses managing adolescent violent behavior will be excluded from the review.

### Population

Although scoping reviews do not primarily aim to evaluate the effectiveness of interventions, the PCC framework was used to provide additional clarification regarding the focus of this review. The population of interest includes nurses or nursing personnel involved in adolescent violence prevention and management within primary health care, community health care, school-based services, or other health care settings.

### Intervention

The included interventions of this scoping review are training and education initiatives that enhance nurses’ competencies regarding the prevention of violence in adolescents. These may include modules on emotional regulation, conflict resolution, early detection of violence risk, and communication strategies for managing adolescents. The training can be delivered through workshops, seminars, simulation-based learning, online courses, or blended methods. Some studies may also include the use of digital communication media as part of training delivery such as mobile apps, web-based platforms, social media, educational games, or interactive tools to support nurses in health promotion, emotional management, and psychosocial education for adolescents.

### Context

The context of this review encompasses primary health care settings, community health care services, school health services, and other health care settings where nurses provide adolescent health services and are involved in violence prevention and management activities.

### Outcomes

Because the objective of this scoping review is to map the breadth of existing evidence, outcomes will not be predefined as eligibility criteria. Instead, all reported outcomes associated with nurse training interventions for adolescent violence prevention will be charted during data extraction. These may include outcomes related to nurses (eg, knowledge, attitudes, competencies, confidence, and self-efficacy), adolescents (eg, behavioral and psychosocial outcomes), and implementation outcomes (eg, feasibility, acceptability, and sustainability).

### Identification of Relevant Studies

A comprehensive literature search will be conducted to identify studies relevant to violence prevention training programs for nurses in the context of adolescent violent behavior management. Electronic databases to be searched include PubMed, Scopus, CINAHL, ProQuest, Web of Science, and ScienceDirect. Gray literature sources, including Google Scholar, institutional repositories, reports, and theses, will also be searched to identify additional relevant evidence. The search strategy is detailed in the next section.

### Search Strategy

The search strategy will be developed in accordance with the objectives and research questions of this scoping review. Searches will be conducted using a combination of controlled vocabulary terms, including MeSH and free-text keywords related to nurses, violence prevention, adolescent violent behavior, and training or educational programs. Reference lists of included studies will also be screened manually to identify additional eligible articles.

Keywords and phrases such as “violence prevention,” “aggression management,” “de-escalation training,” “nurse,” “nursing staff,” “adolescent,” “youth,” and “training program” will be combined using Boolean operators such as AND and OR. Quotation marks will be used for exact phrases where appropriate, and search strategies will be adapted according to the syntax and indexing systems of each database.

Filters and limits applied during the search process will include publication years between 2000 and 2024, articles published in English or Indonesian, and studies available in full text when accessible. The search process and study selection procedure will be presented using a PRISMA (Preferred Reporting Items for Systematic Reviews and Meta-Analyses) flow diagram.

To ensure transparency and reproducibility, the complete PubMed search strategy for one electronic database is provided in Multimedia Appendix 1 and reported according to the PRISMA-S (Preferred Reporting Items for Systematic Reviews and Meta-Analyses Literature Search Extension) recommendations.

### Study Selection

All retrieved records will be imported into EndNote (Clarivate; version 20) and subsequently uploaded into Rayyan (Rayyan Systems Inc; version 2024) for screening and duplicate removal. Two reviewers will independently screen titles and abstracts according to the eligibility criteria. Full-text articles of potentially relevant studies will then be assessed independently by the same reviewers. Disagreements at any stage will be resolved through discussion, and where consensus cannot be reached, a third reviewer will be consulted. The study selection process will be reported using a PRISMA-ScR flow diagram.

### Data Charting and Extraction

A standardized data charting form will be developed to systematically extract and organize relevant information from the included studies. The form will be pilot-tested on a small number of studies and refined iteratively throughout the review process to ensure consistency and relevance to the review objectives and research questions.

The extracted data will include author(s), year of publication, country of study, study design, study objectives, participant characteristics, health care or community setting, characteristics of the training or educational program, theoretical frameworks, delivery methods, duration of intervention, implementation strategies, measurement or assessment tools, and reported findings related to nurses’ competencies in adolescent violence prevention (Table 2). Information related to barriers, facilitators, implementation experiences, and recommendations for practice or future research will also be extracted where available.

**Table 2.** Data extraction variables.

Variable	Description
Study title	Full title of the article
Authors and year	Author(s) and year of publication
Country and context	Study setting, country, and health care/community context
Study design	Qualitative, quantitative, mixed methods, review
Population	Nurses or nursing personnel involved in adolescent violence prevention or care
Participant characteristics	Age group, professional role, sample size (if available)
Type of intervention	Description of training or educational intervention
Theoretical framework	Underlying theories or models (eg, social ecological model, goal attainment theory)
Training method	Mode of delivery (workshop, simulation, online, blended learning, etc)
Duration and frequency	Length and frequency of intervention
Implementation strategy	How the training was delivered and supported
Outcome measures	Nurses’ knowledge, attitudes, skills, self-efficacy, or other reported outcomes
Measurement tools	Instruments or scales used (if available)
Key findings	Main results as reported in the study
Barriers and facilitators	Factors influencing implementation or effectiveness
Study limitations	Limitations reported by authors

Data charting will be conducted independently by two reviewers using the standardized form. Any discrepancies in the extracted data will be resolved through discussion and consensus, with consultation from a third reviewer if necessary. The extracted data will be reviewed regularly to ensure accuracy, completeness, and alignment with the objectives of the scoping review.

### Data Synthesis

Data will be summarized in narrative and tabular forms to map the extent, characteristics, and distribution of the existing evidence regarding violence prevention training programs for nurses in the context of adolescent violent behavior management. Consistent with the purpose of a scoping review, the synthesis will focus on describing and mapping available evidence rather than evaluating intervention effectiveness.

Descriptive analysis (frequencies and percentages) will be conducted for quantitative data where appropriate, while qualitative findings will be synthesized using thematic analysis following the 6-phase approach proposed by Braun and Clarke [42]. The analysis will involve six iterative phases: (1) familiarization with the extracted data, (2) generation of initial codes, (3) searching for potential themes, (4) reviewing and refining themes, (5) defining and naming themes, and (6) producing the narrative synthesis. Themes will be developed inductively to map intervention characteristics, implementation strategies, barriers, facilitators, and reported outcomes related to nurse training for adolescent violence prevention. The synthesis process will also examine the characteristics of training programs, educational components, delivery methods, theoretical frameworks, assessment approaches, and reported findings related to nurses’ competencies in adolescent violence prevention.

The findings will be presented in narrative summaries, evidence tables, and thematic groupings aligned with the objectives and research questions of the review. Quality appraisal will not be performed, as it is not mandatory for scoping reviews and the primary aim of this review is to comprehensively map the existing literature.

### Presentation and Reporting of Results

Results will be presented according to the objectives and research questions of the review, including characteristics of training programs, educational components, delivery methods, theoretical frameworks, assessment approaches, implementation strategies, and reported findings related to nurses’ competencies in adolescent violence prevention. The synthesis will discuss the extent, nature, and limitations of the existing evidence and provide implications for nursing practice, policy, and future research.

The study selection process will be presented using a PRISMA-ScR flow diagram, while the review findings will be summarized in narrative form and supported by evidence tables and thematic groupings where appropriate.

### Consultation Process

Validity of the findings, missing evidence, and interpretation of results will be addressed through an expert consultation involving psychiatric nursing, adolescent psychology, and public health experts. Expert input will be included in the final synthesis to increase the relevance and utility of the review.

### Ethical Considerations

As this is a review study, we use secondary data collected from previously published studies and therefore do not need any formal ethical approval. However, all procedures will be conducted in accordance with research ethics guidelines, such as following citation rules and acknowledging intellectual property rights. If the results are later to be used as the basis for a human study, ethical approval shall be sought before collecting data.

## Results

Literature searches across the selected electronic databases were initiated in March 2025 using predefined search terms. Title and abstract screening, as well as preliminary appraisal of eligible studies, were conducted in May 2026 following the Joanna Briggs Institute methodology and PRISMA-ScR guidelines. Full-text screening, data extraction, and thematic analysis are currently ongoing and are expected to be completed by July 2026. The findings of this scoping review will be submitted to a journal by September 2026.

This scoping review is expected to systematically map and synthesize the existing evidence regarding nurse training programs for the prevention of adolescent violent behavior in primary health care settings. The review findings will be organized in accordance with the objectives and research questions of the study, particularly focusing on the identification of risk and protective factors associated with adolescent violent behavior, the characteristics of nurse training and educational programs, and the theoretical frameworks underpinning these interventions. This scoping review is expected to provide a comprehensive map of existing evidence regarding nurse training interventions for adolescent violence prevention, including characteristics of interventions, theoretical foundations, implementation approaches, and reported outcomes. The findings will inform the development of the Kepolar model and support future nursing practice, policy, and research.

The Kepolar model will subsequently be further developed and tested in the researcher’s (SM) doctoral dissertation. Moreover, the review is expected to contribute to practical and policy recommendations aimed at strengthening the promotive and preventive roles of nurses in *puskesmas* settings, thereby supporting adolescent mental health promotion and violence prevention efforts in Indonesia.

## Discussion

This scoping review is expected to provide a comprehensive mapping of existing evidence regarding nurse training interventions for adolescent violence prevention and management within primary health care and community health care settings. This scoping review protocol concentrates on training targeting primary health care nurses to reduce the risk of violent behavior in adolescents, a direction that turns the focus from interventions exclusively aimed at adolescents toward equipping frontline health workers to be proactive regarding violence prevention. Early results from the international literature discuss that education and training for health care workers, including nurses, can improve confidence, readiness, communication skills, and practical competencies concerning risk assessment and management of violent situations (eg, de-escalation and referral screening) [43]. However, there remains relatively little direct evidence regarding the effectiveness of nurse-targeted training in reducing adolescent violence, with considerable heterogeneity in study designs, intervention characteristics, and outcome measures [26]. The review is also anticipated to demonstrate that evidence specifically focusing on structured nurse-targeted training for adolescent violence prevention remains limited compared with broader school-based or youth-focused violence prevention programs.

Recent evidence underscores the critical role of multilevel interventions (eg, individual, family, school, health care system, and community levels) in accordance with the social ecological model [44]. Several assessed programs have primarily targeted adolescents through school-based life skills education, peer support interventions, and community psychosocial programs, demonstrating improvements in social competence, emotional regulation, knowledge, and risk-taking behaviors. Nevertheless, relatively few studies specifically examine nurse-targeted training, particularly within primary health care settings, where nurses may function as frontline screeners, facilitators, referral coordinators, and providers of psychosocial support. The present scoping review extends prior work by specifically focusing on nurse-targeted training interventions within health care and primary health care settings. This focus is particularly relevant in Indonesia and other low- and middle-income countries, where primary health care centers (*puskesmas*) often function as the first point of contact for adolescent health services and community mental health support.

The literature indicates that training programs incorporating theoretical foundations (eg, trauma-informed care, ecological approaches, and life skills frameworks), ongoing supervision, and interactive learning modalities such as simulation, role-playing, communication exercises, de-escalation training, and case-based learning may result in greater competency acquisition and more sustainable behavioral change among health care providers [32]. In addition, multisession interventions with longer duration may provide more sustained effects compared with single-session workshops [45]. Accordingly, this review may help identify important intervention characteristics, including theoretical underpinnings, training content, delivery methods, implementation strategies, and duration of training programs.

The findings of this review may also help clarify several important competencies and components that should be considered in nurse training programs for adolescent violence prevention and management. Core competencies may include early identification of violence risk factors, communication and therapeutic relationship skills, emotional regulation support, de-escalation techniques, behavioral assessment, trauma-informed care, crisis intervention, family engagement, referral coordination, and interprofessional collaboration. Existing literature also suggests that effective training programs often incorporate interactive and practice-based approaches such as simulation, role-playing, case discussions, and reflective learning activities rather than relying solely on didactic lectures [32,45,46].

In addition, nurse training programs may require adaptation to local health care contexts, particularly within primary health care systems such as *puskesmas* in Indonesia, where nurses frequently manage adolescents with limited mental health resources and multidisciplinary support. Therefore, training programs may benefit from incorporating culturally appropriate approaches, practical referral algorithms, community-based case management strategies, and implementation guidance relevant to resource-limited settings. These findings may support the future development of more structured, competency-based, and contextually appropriate nurse training models for adolescent violence prevention.

Institutional and systemic support including operational guidelines, referral pathways, leadership endorsement, protected implementation time, and intersectoral collaboration between health care services, schools, and child protection agencies have been identified as important determinants of training implementation and effectiveness [35,45]. Therefore, the focus on *puskesmas* in this protocol is considered particularly relevant because primary health care centers may function as coordination hubs connecting health care, educational, family, and community sectors in adolescent violence prevention efforts. However, in the absence of broader structural and organizational support, individual-level training alone may have limited impact. Accordingly, mapping studies that evaluate implementation-related constructs such as feasibility, acceptability, adherence, and sustainability may provide important insights for future intervention development and implementation.

The majority of previous studies have evaluated training outcomes primarily at the provider level, including knowledge, attitudes, confidence, preparedness, and self-efficacy [32,47], while relatively fewer studies have assessed adolescent-level outcomes such as violent incidents, psychosocial functioning, school engagement, school attendance, or mental health indicators. Both provider-level and adolescent-level outcomes are considered within this protocol through the inclusion of studies evaluating nursing competencies as well as intervention impacts on adolescents. Importantly, improvements in nurses’ competencies alone may not necessarily reduce adolescent violence unless broader contextual issues including school culture, policy support, cross-sector partnerships, and health care infrastructure are also addressed [48]. The findings of this scoping review may therefore support policymakers and health care administrators in designing integrated adolescent violence prevention strategies that combine workforce development with broader system-level strengthening.

Current systematic reviews and experimental studies focusing on violence prevention interventions remain methodologically heterogeneous, with many studies characterized by small sample sizes, inconsistent outcome measures, and limited reporting transparency. Among nurse training studies, many employ pre–post or qualitative evaluative designs, requiring cautious interpretation regarding intervention effectiveness. In addition, this review may identify considerable methodological heterogeneity across studies, including differences in study design, intervention components, measurement instruments, and reported outcomes [47]. As such, this scoping review represents an important initial step toward systematically identifying and synthesizing the nature, scope, and characteristics of current evidence before future large-scale intervention and implementation studies are conducted.

Recent research has also explored innovative training modalities, including online learning platforms, blended learning approaches, digital educational modules, and virtual reality simulation training, to improve communication and de-escalation skills among health care workers [46]. Early evidence suggests that these approaches may increase engagement and facilitate skill acquisition, although long-term effectiveness and sustainability remain under investigation [46,48]. Future studies should also consider the use of standardized outcome measures to improve comparability across interventions. Further research is also needed to explore culturally appropriate and context-specific training models applicable to primary health care systems in Indonesia and other low- and middle-income countries.

Several limitations identified in the international literature are also relevant to this protocol, including the limited availability of high-quality evidence specifically evaluating nurse training for adolescent violence prevention, substantial heterogeneity in terminology and outcome measures across studies, and contextual differences requiring adaptation of interventions to local health care systems and resource availability. This scoping review seeks to address some of these limitations through the inclusion of peer-reviewed literature, gray literature, qualitative studies, and instrument validation studies to provide a broader and more comprehensive evidence mapping.

The findings of this scoping review will be disseminated through publication in peer-reviewed journals, presentations at national and international scientific conferences, and communication with health care stakeholders and policymakers. Dissemination activities may also include practical outputs such as stakeholder discussions, educational materials, implementation recommendations, policy briefs, and evidence-informed guidance to facilitate the translation of findings into nursing practice and adolescent mental health services. This scoping review protocol also has several strengths. It follows internationally recognized methodological frameworks, including the Joanna Briggs Institute methodology and Arksey and O’Malley framework, and includes both peer-reviewed and gray literature to support comprehensive evidence mapping. Another strength of this review is its focus on adolescent violence prevention within primary health care contexts, an area that remains relatively underexplored in existing literature. However, as a scoping review, this study will not formally assess the methodological quality or risk of bias of included studies. In addition, heterogeneity across study designs, intervention approaches, and outcome measures may limit comparability across studies.

This scoping review protocol aims to systematically map and synthesize evidence regarding nurse training interventions for adolescent violence prevention and management within primary health care settings. Ultimately, this review may support future research, nursing practice, and policy development related to adolescent violence prevention within community and primary health care systems.

## Supplementary material

10.2196/89576 Multimedia Appendix 1 Full PRISMA-S search strategies.

10.2196/89576 Checklist 1 PRISMA-ScR checklist.

## Abbreviations

Kepolar: *Keterampilan Pencegahan Risiko Perilaku Kekerasan Remaja* (Prevention of Adolescent Violence-Risk Behavior)
MeSH: Medical Subject Headings
PCC: Population-Concept-Context
PRISMA: Preferred Reporting Items for Systematic Reviews and Meta-Analyses
PRISMA-S: Preferred Reporting Items for Systematic Reviews and Meta-Analyses Literature Search Extension
PRISMA-ScR: Preferred Reporting Items for Systematic Reviews and Meta-Analyses Extension for Scoping Reviews
UNESCO: United Nations Educational, Scientific and Cultural Organization
