# Supplementary material for: Utilization of Training for Nurses in the Prevention of Adolescent Violence Risk Behaviors: Protocol for a Scoping Review
Source: JMIR Res Protoc. 2026 Aug 10;15:e89576. doi: 10.2196/89576 (PMC13456303; doi:10.2196/89576)
Supplement: Supplementary file 1 [file resprot-v15-e89576-s001.docx]

**Appendix 1. Full PRISMA-S Search Strategies**

**PubMed (MEDLINE)**

("Nurses"[Mesh] OR nurse* OR "nursing staff" OR "nursing personnel")
AND
("Adolescent"[Mesh] OR adolescen* OR teen* OR youth OR "young people")
AND
("Violence"[Mesh] OR violence OR aggression OR "aggressive behavior" OR bullying OR "externalizing behavior" OR "violent behavior")
AND
("Education"[Mesh] OR "Inservice Training"[Mesh] OR training OR education OR "training program*" OR workshop* OR simulation OR "de-escalation training" OR "capacity building")

Filters applied:

- Publication years: 2000–2024
- Language: English, Indonesian

**Scopus**

(TITLE-ABS-KEY(nurse* OR "nursing staff" OR "nursing personnel"))
AND
(TITLE-ABS-KEY(adolescen* OR teen* OR youth OR "young people"))
AND
(TITLE-ABS-KEY(violence OR aggression OR "aggressive behavior" OR bullying OR "violent behavior" OR "externalizing behavior"))
AND
(TITLE-ABS-KEY(training OR education OR "training program*" OR workshop* OR simulation OR "de-escalation training" OR "capacity building"))

Filters applied:

- Year range: 2000–2024

**ProQuest**

AB(nurse* OR "nursing staff" OR "nursing personnel")
AND
AB(adolescen* OR teen* OR youth OR "young people")
AND
AB(violence OR aggression OR "aggressive behavior" OR bullying OR "violent behavior")
AND
AB(training OR education OR "training program*" OR workshop* OR simulation OR "de-escalation training")

Filters applied:

- Publication years: 2000–2024
- Source types: scholarly journals, dissertations, reports
- Language: English, Indonesian

**Cochrane Library**

(nurse* OR "nursing staff" OR "nursing personnel")
AND
(adolescen* OR teen* OR youth OR "young people")
AND
(violence OR aggression OR "aggressive behavior" OR bullying OR "violent behavior")
AND
(training OR education OR "training program*" OR workshop* OR simulation OR "de-escalation training")

Limits applied:

- Publication years: 2000–2024
- Trials and systematic reviews

**Grey Literature (Google Scholar / GreyNet)**

**Google Scholar**

nurse training adolescent violence prevention education simulation de-escalation

Screening limits:

- Years: 2000–2024
- First 200–300 results screened
- English or Indonesian language

**GreyNet**

nurse* AND adolescent AND violence AND training
