# Supplementary material for: Utilization of Training for Nurses in the Prevention of Adolescent Violence Risk Behaviors: Protocol for a Scoping Review
Source: JMIR Res Protoc. 2026 Aug 10;15:e89576. doi: 10.2196/89576 (PMC13456303; doi:10.2196/89576)
Supplement: Supplementary file 2 [file resprot-v15-e89576-s002.docx]

Appendix 2: PRISMA-ScR Checklist

| Section / Topic | PRISMA-ScR Item | Description in Protocol | Yes | No |
| --- | --- | --- | --- | --- |
| ADMINISTRATIVE  INFORMATION |  |  |  |  |
| Title | 1a. Identify the report as a scoping review  protocol. | The title clearly states “Protocol for a Scoping  Review.” | ✓ |  |
|  | 1b. If this is an update of a previous protocol, state  so. | Not applicable. |  | ✓ |
| Registration | 2. Indicate the name of the registry and  registration number. | Will be registered in the Open Science Framework (OSF). | ✓ |  |
| Authors | 3a. Provide names, affiliations, and contact information for the  corresponding author. | Listed on the title page. | ✓ |  |
|  | 3b. Describe author  contributions. | Described in the contributor  section. | ✓ |  |
| Amendments | 4. Explain how protocol amendments will be  documented. | No amendments at this initial stage. |  | ✓ |
| Support / Funding | 5a. Identify sources of  funding or support. | Self-funded by the  researchers. | ✓ |  |
|  | 5b. Name the supporting  institution or sponsor. | Faculty of Nursing,  Universitas Sriwijaya. | ✓ |  |
|  | 5c. Describe the sponsor’s  role in protocol preparation. | No conflict of interest. | ✓ |  |
| INTRODUCTION |  |  |  |  |
| Rationale | 6. Describe the rationale and context for conducting the scoping  review. | Stated in the Background, highlighting nurses’ roles in preventing adolescent  violence. | ✓ |  |
| Objectives | 7. Present the purpose and focus of the review using the PCC (Population, Concept,  Context) framework. | Explained in the Objectives section, covering population (nurses), concept (training), and context (adolescent  violence prevention). | ✓ |  |

| METHODS |  |  |  |  |
| --- | --- | --- | --- | --- |
| Eligibility Criteria | 8. Define inclusion and  exclusion criteria. | Clearly described using the  PCC framework. | ✓ |  |
| Information Sources | 9. Specify databases and sources of grey literature. | PubMed, Scopus, ProQuest,  Cochrane Library, Google Scholar, and GreyNet. | ✓ |  |
| Search Strategy | 10. Provide a reproducible search  strategy. | Explained with keyword combinations and Boolean  operators. | ✓ |  |
| STUDY RECORDS |  |  |  |  |
| Data Management | 11a. Describe how data will be managed. | Mendeley and Microsoft Excel will be used for reference management and  data extraction. | ✓ |  |
| Selection Process | 11b. Explain how studies will be selected by independent reviewers. | Two reviewers will independently screen studies, with verification by a  third reviewer. | ✓ |  |
| Data Collection Process | 11c. Describe data collection and verification  procedures. | Data will be extracted in duplicate and consolidated  through discussion. | ✓ |  |
| Data Items | 12. List variables to be extracted. | Study type, participant characteristics, type and method of training, duration,  and measured outcomes. | ✓ |  |
| Outcomes and  Prioritization | 13. Define main and  additional outcomes. | Knowledge, attitudes, skills,  and self-efficacy of nurses. | ✓ |  |
| Risk of Bias | 14. Explain any approach for assessing risk of bias. | Not conducted, as this is a scoping review rather than a  systematic review. | ✓ |  |
| RESULTS SYNTHESIS |  |  |  |  |
| Data Synthesis | 15. Describe how the data  will be synthesized. | Descriptive and thematic  analysis will be applied. | ✓ |  |
| Meta-bias(es) | 16. Specify any meta-bias  if applicable. | Not applicable for a scoping  review. |  | ✓ |
| Confidence in Evidence | 17. Explain assessment of evidence strength if  performed. | Not applicable. |  | ✓ |

| CONSULTATION | 18. Describe plans for consultation with experts or stakeholders. | Will involve experts in psychiatric nursing, public health, and local health  authorities. | ✓ |  |
| --- | --- | --- | --- | --- |
| ETHICAL CONSIDERATIONS | 19. Explain ethical aspects of the study. | Ethical approval is not required as secondary data will be used; academic  integrity will be upheld. | ✓ |  |
